# Integrating multiple lines of evidence to assess the effects of maternal BMI on pregnancy and perinatal outcomes

**DOI:** 10.1186/s12916-023-03167-0

**Published:** 2024-01-29

**Authors:** Maria Carolina Borges, Gemma L. Clayton, Rachel M. Freathy, Janine F. Felix, Alba Fernández-Sanlés, Ana Gonçalves Soares, Fanny Kilpi, Qian Yang, Rosemary R. C. McEachan, Rebecca C. Richmond, Xueping Liu, Line Skotte, Amaia Irizar, Andrew T. Hattersley, Barbara Bodinier, Denise M. Scholtens, Ellen A. Nohr, Tom A. Bond, M. Geoffrey Hayes, Jane West, Jessica Tyrrell, John Wright, Luigi Bouchard, Mario Murcia, Mariona Bustamante, Marc Chadeau-Hyam, Marjo-Riitta Jarvelin, Martine Vrijheid, Patrice Perron, Per Magnus, Romy Gaillard, Vincent W. V. Jaddoe, William L. Lowe, Bjarke Feenstra, Marie-France Hivert, Thorkild I. A. Sørensen, Siri E. Håberg, Sylvain Serbert, Maria Magnus, Deborah A. Lawlor

**Affiliations:** 1 MRC Integrative Epidemiology Unit at the University of Bristol, Oakfield House, Oakfield Grove, Bristol, BS8 2BN UK; 2 Population Health Science, Bristol Medical School, University of Bristol, Bristol, UK; 3 Institute of Biomedical and Clinical Science, College of Medicine and Health, University of Exeter, Exeter, UK; 4 The Generation R Study Group, Erasmus MC, University Medical Center Rotterdam, Rotterdam, The Netherlands; 5 Department of Pediatrics, Erasmus MC, University Medical Center Rotterdam, Rotterdam, The Netherlands; 6 Bradford Institute for Health Research, Bradford Teaching Hospitals NHS Trust, Bradford, UK; 7 Department of Epidemiology Research, Statens Serum Institut, Copenhagen, Denmark; 8 Department of Preventive Medicine and Public Health, University of the Basque Country, Leioa, Spain; 9 BIODONOSTIA Health Research Institute, Paseo Dr. Beguiristain, 20014 San Sebastian, Spain; 10 CIBER Epidemiología y Salud Pública (CIBERESP), Madrid, Spain; 11 MRC Centre for Environment and Health, School of Public Health, Imperial College London, London, UK; 12 Department of Preventive Medicine, Northwestern University Feinberg School of Medicine, Chicago, IL USA; 13 Institute of Clinical Research, University of Southern Denmark, Odense, Denmark; 14 Department of Epidemiology and Biostatistics, Imperial College London, London, UK; 15 The University of Queensland Diamantina Institute, The University of Queensland, Brisbane, Australia; 16 Division of Endocrinology, Metabolism, and Molecular Medicine, Department of Medicine, Northwestern University Feinberg School of Medicine, Chicago, IL USA; 17 Department of Biochemistry and Functional Genomics, Faculty of Medicine and Health Sciences, Université de Sherbrooke, Sherbrooke, Québec Canada; 18 Epidemiology and Environmental Health Joint Research Unit, FISABIO-Universitat Jaume I-Universitat de València, Valencia, Spain; 19 ISGlobal, Institute for Global Health, Barcelona, Spain; 20 Universitat Pompeu Fabra (UPF), Barcelona, Spain; 21 Faculty of Medicine, School of Public Health, Imperial College London, London, UK; 22 Centre de Recherche du Centre Hospitalier Universitaire de Sherbrooke (CR-CHUS), Sherbrooke, Québec Canada; 23 Department of Medicine, Faculty of Medicine and Health Sciences, University of Sherbrooke, Sherbrooke, Québec Canada; 24 Centre for Fertility and Health, Norwegian Institute of Public Health, Oslo, Norway; 25 Department of Population Medicine, Harvard Medical School, Harvard Pilgrim Health Care Institute, Boston, MA USA; 26 Diabetes Unit, Massachusetts General Hospital, Boston, MA USA; 27 Department of Public Health, Section of Epidemiology, Faculty of Health and Medical Sciences, University of Copenhagen, Copenhagen, Denmark; 28 Novo Nordisk Foundation Center for Basic Metabolic Diseases, Faculty of Health and Medical Sciences, University of Copenhagen, Copenhagen, Denmark; 29 Center For Life-Course Health Research, Faculty of Medicine, University of Oulu, Oulu, Finland

**Keywords:** Pregnancy, Body mass index, Triangulation, Mendelian randomisation

## Abstract

**Background:**

Higher maternal pre-pregnancy body mass index (BMI) is associated with adverse pregnancy and perinatal outcomes. However, whether these associations are causal remains unclear.

**Methods:**

We explored the relation of maternal pre-/early-pregnancy BMI with 20 pregnancy and perinatal outcomes by integrating evidence from three different approaches (i.e. multivariable regression, Mendelian randomisation, and paternal negative control analyses), including data from over 400,000 women.

**Results:**

All three analytical approaches supported associations of higher maternal BMI with lower odds of maternal anaemia, delivering a small-for-gestational-age baby and initiating breastfeeding, but higher odds of hypertensive disorders of pregnancy, gestational hypertension, preeclampsia, gestational diabetes, pre-labour membrane rupture, induction of labour, caesarean section, large-for-gestational age, high birthweight, low Apgar score at 1 min, and neonatal intensive care unit admission. For example, higher maternal BMI was associated with higher risk of gestational hypertension in multivariable regression (OR = 1.67; 95% CI = 1.63, 1.70 per standard unit in BMI) and Mendelian randomisation (OR = 1.59; 95% CI = 1.38, 1.83), which was not seen for paternal BMI (OR = 1.01; 95% CI = 0.98, 1.04). Findings did not support a relation between maternal BMI and perinatal depression. For other outcomes, evidence was inconclusive due to inconsistencies across the applied approaches or substantial imprecision in effect estimates from Mendelian randomisation.

**Conclusions:**

Our findings support a causal role for maternal pre-/early-pregnancy BMI on 14 out of 20 adverse pregnancy and perinatal outcomes. Pre-conception interventions to support women maintaining a healthy BMI may reduce the burden of obstetric and neonatal complications.

**Funding:**

Medical Research Council, British Heart Foundation, European Research Council, National Institutes of Health, National Institute for Health Research, Research Council of Norway, Wellcome Trust.

**Supplementary Information:**

The online version contains supplementary material available at 10.1186/s12916-023-03167-0.

## Background

Obesity is a leading preventable cause of ill health, mortality, and morbidity across the world and affects 10% and 25% of adult women in low- and high-income countries, respectively [1]. Higher maternal pre-pregnancy body mass index (BMI) is associated with a higher risk of various adverse pregnancy and perinatal outcomes, including pregnancy loss, gestational hypertension (GH), preeclampsia (PE), gestational diabetes mellitus (GDM), perinatal depression, caesarean deliveries, preterm birth (PTB), large for gestational age (LGA), and no breastfeeding initiation [2–12]. However, given the ethical and logistical challenges of conducting randomised controlled trials (RCTs) in pregnancy, most evidence in the field comes from conventional observational studies, which may be confounded by unmeasured or inaccurately measured maternal characteristics, such as socioeconomic position, age, parity, ethnicity, smoking, and alcohol intake.

Understanding the impact of maternal pre-pregnancy BMI on pregnancy and perinatal health is key to inform appropriate interventions aimed at preventing adverse outcomes and to predict their future burden in different populations. A better understanding of the potential causal role of BMI can be achieved by integrating multiple lines of evidence in a triangulation framework [13, 14], which can help overcome fundamental biases arising from the reliance on a single method (e.g. multivariable regression in observational studies). In this context, more credible causal inference can be made for findings in agreement across different analytical approaches with different strengths and limitations; while disagreement could decrease confidence in previous findings or highlight specifics of future research needs, for example where there is imprecision in results from some approaches.

**Fig. 1:**
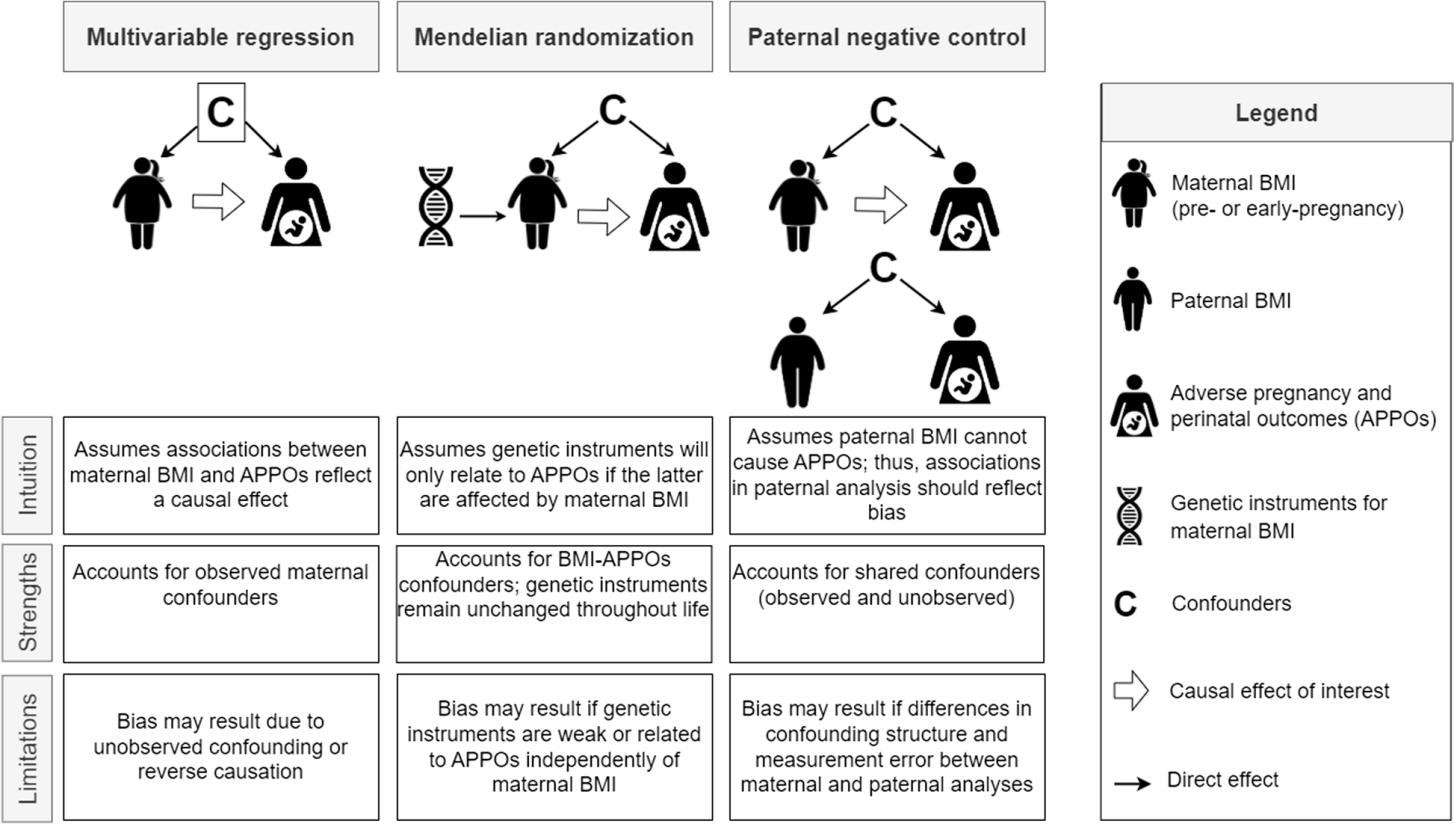
Overview of the three analytical approaches used to investigate the effect of maternal body mass index on adverse pregnancy and perinatal outcomes. A brief description of each approach is presented in the context of exploring the effect of maternal BMI on APPOs’ risk. Given each approach has different strengths and limitations, findings that agree across approaches are likely to be more credible. The description of each approach is simplified for illustration purposes. An extensive description of assumptions and sources of bias for each approach has been reported previously (e.g. [17–21]). The box around the confounders in the multivariable regression reflects the assumption of the method that all confounders were accurately adjusted for in the analyses. BMI, body mass index; APPOs, adverse pregnancy and perinatal outcomes

The aim of this study was to explore the relation of maternal pre-/early pregnancy BMI (hereafter ‘maternal BMI’) with a wide range of pregnancy and perinatal outcomes by integrating evidence from multivariable regression, Mendelian randomisation, and paternal negative control. The combination of these three approaches provides a unique contribution to the evidence basis on the causal effect of maternal BMI given their different strengths and limitations. While findings from conventional observational studies using multivariable regression might be biased by residual confounding, Mendelian randomisation studies are less prone to such form of confounding but may be biased by weak instruments or unbalanced horizontal pleiotropy [15, 16]. The use of negative control designs, such as using paternal BMI as a negative control exposure, can reveal bias in associations of maternal BMI with adverse pregnancy and perinatal outcomes since paternal BMI is unlikely to affect these outcomes, but may be associated with unmeasured confounders in a similar way to maternal BMI (Fig. 1) [17, 18].

## Methods

### Study participants

Data were obtained from up to 446,526 women participating in 14 studies in Europe and North America as part of the MR-PREG collaboration [22] (Table 1). We included women who had available information on at least one outcome of interest, had a singleton birth, delivered a baby without a severe known congenital anomaly, and were of European ancestry since most studies included participants of European descent only or predominantly. Informed consent was obtained from all participants and study protocols were approved by the local, regional, or institutional ethics committees. Details of recruitment, data collection, and ethical approval of each study can be found in Additional file 1: Supplementary Methods [23–55].

**Table 1:** Characteristics of the included studies

Cohort	Source	Country	Year	Maximum* N*^a^	BMI measurement	Maternal pre-pregnancy BMI [kg/m^2^]^b^ Mean (SD)	Maternal age at delivery [years] Mean (SD)
ALSPAC*	[23, 24]	UK	1991–1992	11,272	Self-reported pre-pregnancy	22.95 (3.82)	28.46 (4.78)
BiB*	[26]	UK	2007–2010	5018	Measure around 12 weeks of gestation	26.63 (5.99)	26.82 (5.96)
DNBC-GOYA*	[28, 29]	Denmark	1996–2002	2542	Self-reported pre-pregnancy	23.59 (4.30)	29.67 (4.20)
DNBC-PTB (controls)*	[32]	Denmark	1996–2002	1676	Self-reported pre-pregnancy	23.44 (3.98)	29.78 (4.10)
EFSOCH*	[34]	UK	2000–2004	789	Weight self-reported pre-pregnancy, height measured during pregnancy	24.02 (4.43)	30.64 (5.03)
FinnGen	[37]	Finland	1969–2018	190,879	NA	NA	NA
GEN-3G	[56]	Canada	2010–2013	582	Self-reported pre-pregnancy	25.04 (5.70)	28.27 (4.34)
GenR	[41]	Netherlands	2002–2006	4138	Measured before 20 weeks of gestation	25.31 (4.89)	28.50 (5.66)
HAPO*	[43]	USA	1999–2002	1310	Measured between 24 and 32 weeks of gestation	28.46 (4.82)	31.31 (5.27)
INMA	[46]	Spain	1997–2011	1035	Self-reported pre-pregnancy weight, height measured in the first trimester	23.37 (4.25)	30.72 (4.02)
MoBa*	[47, 57]	Norway	1999–2008	81,795	Self-reported at 15 weeks gestation	24.05 (4.32)	30.13 (4.72)
NFBC1966	[49]	Finland	1966	356	Self-reported pre-pregnancy	25.14 (4.78)	30.22 (5.9)
NFBC1986	[51]	Finland	1986	883	Self-reported pre-pregnancy	24.17 (4.61)	25.43 (2.64)^c^
UK Biobank	[58]	UK	2006–2010	153,543	NA	NA	29.03 (6.34)^d^

*Abbreviations: ALSPAC* Avon Longitudinal Study of Parents and Children, *BiB* Born in Bradford, *DNBC-GOYA* Danish National Birth Cohort-Genetics of Obesity in Young Adults Study, *DNBC-PTB* Danish National Birth Cohort-Preterm Birth Study, *EFSOCH* Exeter Family Study of Childhood Health, *FinnGen* FinnGen (release 8), *GEN-3G* Genetics of Glycaemic Regulation in Gestation and Growth, *GenR* Generation R, *HAPO* Hyperglycaemia and Adverse Pregnancy Outcome, *INMA* Infancia y Medio Ambiente (English translation = Childhood and the Environment), *MoBa* Norwegian Mother, Father and Child Cohort Study, *NA* Not available, *NFBC1966* Northern Finland 1966 Birth Cohort, *NFBC1986* Northern Finland 1986 Birth Cohort

^*^Studies contributing to Mendelian randomisation analyses adjusted by offspring genotype

^a^Maximum number of mothers with data on at least one outcome

^b^Maternal BMI is only reported where collected pre- or early in pregnancy

^c^The relatively young age at delivery in the NFBC1986 cohort may be explained by the young age of the cohort at the time of the study

^d^Maternal age in UK Biobank was taken from maternity record linkage on a subsample of participants and may therefore not be representative of the full sample included

### Exposure measures

Maternal BMI in kg/m^2^ was calculated from measured or self-reported weight and height data (Table 1). Weight data was collected before pregnancy in eight studies, before 20 weeks of gestation in three studies, and between 24 and 32 weeks of gestation in one study. Two studies did not have a measure of pre- or early-pregnancy BMI and could only contribute to the Mendelian randomisation analyses.

### Outcomes measures

We focused on 20 a priori selected (based on clinical relevance and consensus amongst the study team) binary outcomes: miscarriage, stillbirth, hypertensive disorders of pregnancies (HDP), GH, PE, GDM, maternal anaemia, perinatal depression, pre-labour membrane rupture, induction of labour, caesarean section, PTB, LGA, small-for-gestational age (SGA), low birthweight, high birthweight, low Apgar score after 1 min, low Apgar score after 5 min, neonatal intensive care unit (NICU) admission, and breastfeeding initiation (see Table 2 for definitions and total sample sizes). We included related traits amongst the selected outcomes to maximise the number of cohorts contributing to the analyses (e.g. studies that did not have data on gestational age could contribute with information on low birthweight but not SGA). In additional analyses, we examined four continuous traits that underlie some of these outcomes (i.e. birthweight, birth length, ponderal index at birth, and gestational age at birth). Details on outcomes definitions, distributions, and sample sizes for each contributing study are available in Additional file 1: Supplementary Methods [23–55] and Additional file 2: Supplementary Tables 1A and B.

**Table 2:** Case definition and sample size for pregnancy and perinatal outcomes across participating studies

Outcomes	Case definition	*N*	*N* cases	% cases
*Binary outcomes*				
Miscarriage^a^	Self-reported in index pregnancy	91,757	107	0.12%
	Self-reported in previous pregnancies	376,434	70,181	15.71%
Stillbirth^a^	Self-reported in index pregnancy	91,942	292	0.32%
	Self-reported in previous pregnancies	174,440	4613	2.58%
Hypertensive disorders of pregnancy	Gestational hypertension or preeclampsia^b^	416,803	26,867	6.06%
Gestational hypertension	Elevated blood pressure without proteinuria^b^	406,103	17,607	4.16%
Preeclampsia	Elevated blood pressure with proteinuria^b^	401,184	9827	2.39%
Gestational diabetes	Hyperglycaemia first diagnosed in pregnancy^c^	446,526	14,338	3.11%
Maternal anaemia	Maternal anaemia during pregnancy defined as Hb < 110 g/L (1st trimester) or Hb < 105 g/L(2^nd^ or 3^rd^ trimesters)	92,002	2425	2.57%
Perinatal depression	Self-reported diagnosis or assessed depression symptom scales	113,614	9320	7.58%
Pre-labour rupture of membranes*	Membrane rupture before the onset of contractions	249,265	19,339	7.20%
Induction of labour*	Labour needed induction	114,075	17,351	13.20%
Caesarean section*	Delivery by caesarean section	204,093	27,967	12.05%
Preterm birth*	Gestational age at birth < 37 weeks	261,473	14,090	5.11%
Large-for-gestational age*	> 90^th^ percentile for z-score of birthweight accounting for sex and gestational age^d^	118,667	12,386	9.45%
Small-for-gestational age*	< 10^th^ percentile for z-score of birthweight accounting for sex and gestational age^d^	118,667	8958	7.02%
Low birthweight*	Birthweight < 2500 g	247,716	14,964	5.70%
High birthweight*	Birthweight ≥ 4000 g	239,460	8142	3.29%
Low Apgar score at 1 min*	Apgar score at 1 min < 7	98,868	5760	5.51%
Low Apgar score at 5 min*	Apgar score at 5 min < 7	99,434	1167	1.16%
Neonatal intensive care unit (NICU) admission*	Neonate admitted to NICU	93,522	8262	8.12%
Breastfeeding initiation*	Ever breastfed	94,116	78,472	45.47%
*Continuous outcomes*				
Birthweight*	NA	326,537	NA	NA
Birth length*	NA	95,649	NA	NA
Ponderal index at birth*	NA	95,562	NA	NA
Gestational age at birth*	NA	118,723	NA	NA

Detailed information on each of the outcomes in each cohort is provided in Additional file 1: Supplementary material and Additional file 2: Supplementary table 1

^a^Miscarriage and stillbirths in the index pregnancy were used in multivariable regression and paternal negative control analyses, while miscarriage and stillbirth reported in previous pregnancies were used in Mendelian randomisation analysis

^b^Where possible, we applied the International Society for the Study of Hypertension in Pregnancy criteria (ISSHP), which defines any HDP as SBP ≥ 140 mmHg or DBP ≥ 90 mmHg, measured on two occasions after 20 weeks’ gestation, with those who are then defined as having pre-eclampsia also having proteinuria (with the raised blood pressure) of at least 30 g/Dl and those defined as having gestational hypertension being those who do not meet criteria for pre-eclampsia. By contrast, in some studies (e.g. UK Biobank), information on diagnosis was extracted directly from medical records

^c^Criteria used to define hyperglycaemia first diagnosed in pregnancy varies across studies. Most studies obtained information from questionnaires (i.e. self-reported diagnosis) or from medical records [ICD-10 code O24]

^d^Different reference populations were used to calculate percentiles across studies

^*^For these a priori selected outcomes, additional Mendelian randomisation analyses were conducted accounting for offspring genotype

### Covariables

The following were a priori considered potential confounders of the association between maternal BMI and the pregnancy and perinatal outcomes: maternal age, parity, education, smoking during pregnancy, and alcohol use during pregnancy. We also adjusted for offspring sex to improve statistical efficiency given its strong association with some outcomes (e.g. birthweight-related outcomes). Details of the distribution of these covariables in each study are provided in Additional file 2: Supplementary Table 2.

### Statistical analyses

All analyses were conducted using Stata version 17 (StataCorp, College Station, TX) [59] or R version 4.2.1 (R Foundation for Statistical Computing, Vienna, Austria) [60]. Results are presented as odds ratio (OR) for each binary outcome per standard deviation (SD) increase in maternal BMI to facilitate the comparison of results. The analytical code is available at: https://github.com/gc13313/matbmi_preg.

#### Multivariable regression analyses

In the main analyses, we used logistic regression with two sets of adjustments: (1) maternal age and offspring sex and (2) additionally maternal education, parity, smoking during pregnancy, and alcohol use during pregnancy where available. We present the fully adjusted model as the main analyses and include the minimally adjusted model in the supplementary material. Similar multivariable linear regression models were used for the additional analyses with continuously measured outcomes. Study-specific results were combined using fixed-effects metanalyses (inverse-variance weighted) for the main analyses assuming that there is one true effect size underlying all included studies, and random-effects metanalyses (*DerSimonian and Laird method*) for sensitivity analyses.

#### Mendelian randomisation analysis

**Fig. 2:**
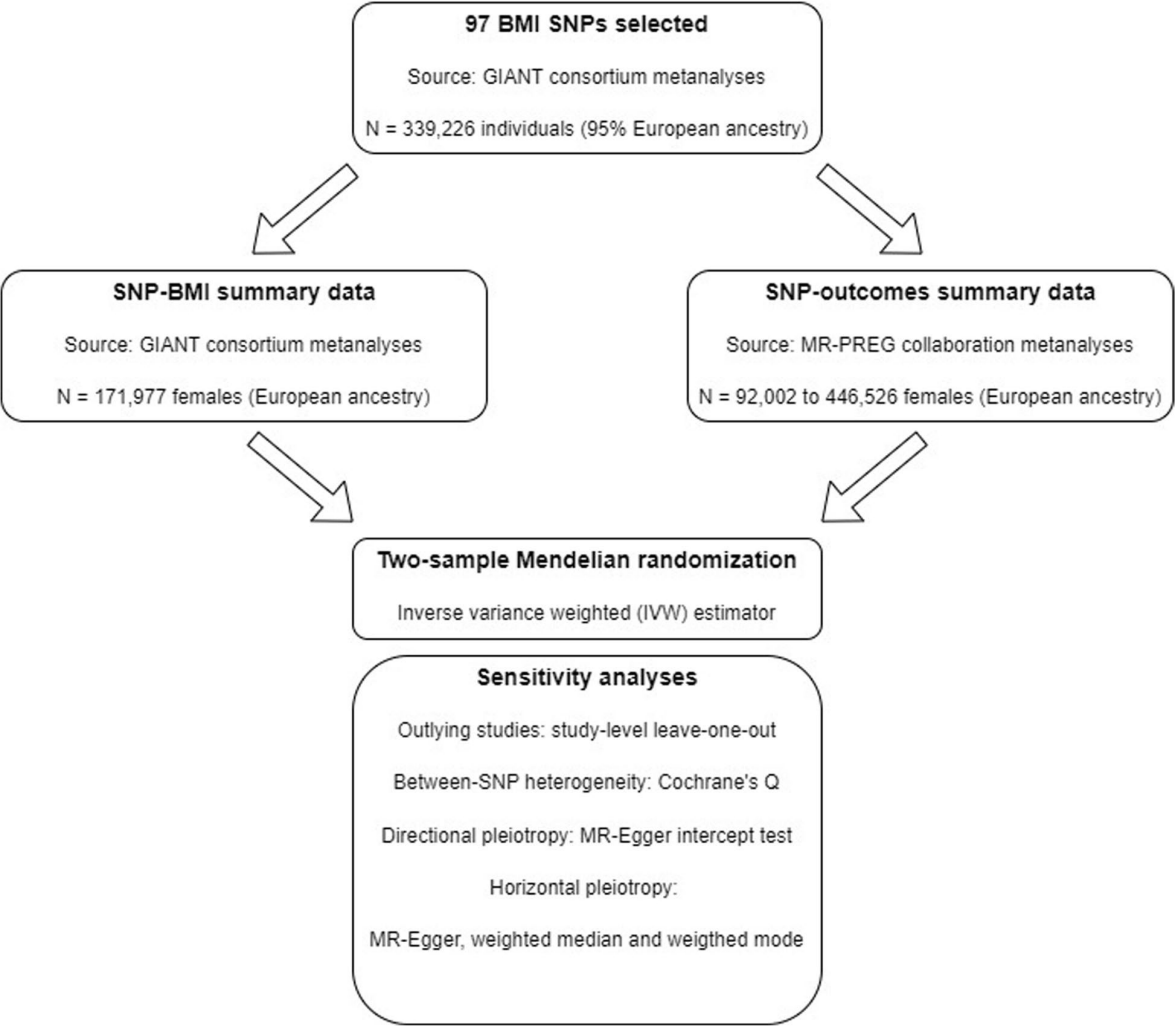
Overview of the two-sample Mendelian randomisation analyses framework. We selected 97 SNPs as instruments for maternal BMI from a genome-wide association studies (GWAS) metanalysis conducted by the Genetic Investigation of ANthropometric Traits (GIANT) consortium [62, 63], including 339,226 males and females. For the selected SNPs, we extracted summary data for the SNP-BMI associations from the GIANT GWAS metanalyses of European ancestry females (*N* = 171,977) and SNP-outcomes associations from European ancestry females from the MR-PREG collaboration (*N* range = 92,002 to 446,526). After harmonising SNP-BMI and SNP-outcomes’ summary data, two-sample MR analyses were carried out using the inverse variance weighted (IVW) method, and a series of sensitivity analyses was performed to assess the plausibility of the core Mendelian randomisation assumptions as specified in the figure. For two studies (Generation R and INMA), summary data was only available to us for 32 SNPs reported in an earlier GIANT BMI GWAS [63], of which 12 SNPs overlapped with the 97 selected SNPs and were included in our metanalyses

We used two-sample Mendelian randomisation, in which the effect of interest is estimated by combining summary data for the association of single nucleotide polymorphisms (SNPs) with BMI and with each outcome, as summarised in Fig. 2 [61]. This approach allowed us to maximise statistical power by including all 14 studies in the analyses even when data on maternal BMI was not available (i.e. FinnGen and UK Biobank).

We selected 97 SNPs previously reported to be strongly associated with BMI (*P* < ${5 x 10}^{-8}$) from a genome-wide association studies (GWAS) metanalysis conducted by the Genetic Investigation of ANthropometric Traits (GIANT) consortium (Additional file 2: Supplementary Table 3) [62]. Unlike more recent BMI GWAS [64], the cohorts included in this GWAS were largely independent from the studies included in our analyses avoiding potential biases due to sample overlap [65, 66].

Summary data for the SNP-BMI associations were obtained from the GIANT GWAS metanalyses of European females (Additional file 2: Supplementary table 3) [62], which included up to 171,977 women (~ 0.5% of participants were also included in our study). We estimated the strength of the genetic instruments using the mean F-statistic and total *R*^2^ for the SNP-BMI association in the GIANT GWAS results as previously described [67, 68]. We also examined the correlation between SNP-BMI estimates in non-pregnant (data from the GIANT consortium) and pregnant women (data from participating cohorts where information on maternal BMI was available to us).

Summary data for the SNP-outcomes associations were obtained from each contributing study using logistic (or linear) regression assuming an additive model. For each SNP, we meta-analysed cohort-specific SNP-outcome associations using inverse-variance weighted fixed-effects for the main analyses and random effects (*DerSimonian and Laird method*) for sensitivity analyses.

The main two-sample MR analyses were carried out using the inverse variance weighted (IVW) method [67]. In addition, we also conducted a leave-one-out analysis at the study level where the pooled IVW estimates were re-computed removing one study at a time to check whether pooled results were driven by a single study.

We conducted a series of sensitivity analyses to explore the plausibility of the core Mendelian randomisation assumption that any effect of SNPs on the outcomes is fully mediated by maternal BMI. We explored the potential presence of invalid instruments (e.g. due to SNPs affecting the outcomes through pathways not mediated by BMI) by (i) assessing between-SNP heterogeneity and directional pleiotropy in effect estimates using Cochran’s Q-statistic and the MR-Egger intercept test [68], respectively, and (ii) using other Mendelian randomisation methods that are more robust to invalid instruments than IVW (MR-Egger [68], weighted median [69], and weighted mode [70]). For offspring outcomes (Table 2), we explored whether IVW estimates might be biased by genetic confounding since maternal BMI genetic variants might influence offspring outcomes (e.g. birthweight) due to the foetus inheriting these variants from the mother rather than due to a causal effect of maternal BMI on the intra-uterine environment [71–73]. This was done by repeating the IVW analyses using summary data for the SNP-outcomes associations adjusted for offspring genotype, which were obtained by regressing each outcome on the maternal genotype for each SNP including the offspring genotype for the respective SNP as a covariable in the model (all genotypes were coded as the number of BMI-increasing alleles).

#### Paternal negative control analyses

We used paternal BMI as a negative control exposure to explore whether the associations of maternal BMI with pregnancy and perinatal outcomes could be explained by residual confounding due to shared familial environment influencing BMI in both partners [18, 74]. These analyses included paternal BMI data from ALSPAC (*N* = 2821–6952), calculated from weight and height self-reported by the father during the first trimester; GenR (*N* = 596–911), measured during the first trimester; and MoBa (*N* = 39,243–57,170), reported by the mother at 15 weeks of gestation. We used multivariable regression to estimate the association of paternal BMI with the outcomes of interest adjusting (where available) for paternal age, number of children, education, smoking, and alcohol intake around the time of their partners’ pregnancy, as well as their partners’ BMI to account for the correlation between maternal and paternal BMI due to assortative mating or shared lifestyle [74, 75] (correlation coefficients ranging from 0.17 in ALSPAC to 0.24 in MoBa). Results were then contrasted between the mutually adjusted maternal and paternal BMI (negative control) analyses. The adjusted maternal regression estimates used for comparison with paternal BMI associations in the negative control analysis differ from the multivariable regression estimates used in the main analysis (that are compared to the Mendelian randomisation estimates). In the paternal negative control comparison, the maternal regression estimates were additionally adjusted for paternal BMI and paternal confounders and therefore restricted to studies reporting both maternal and paternal BMI. Similar estimates between maternal and paternal BMI analyses indicate maternal BMI is unlikely to be a cause of pregnancy and perinatal outcomes via intrauterine mechanisms assuming comparable sources of biases. Conversely, associations that are specific or stronger in the maternal compared to the paternal BMI analyses would support a causal effect of maternal BMI.

### Patient and public involvement

The current research was not informed by patient and public involvement because it used secondary data. This means that patients and the public were not involved in setting the research question or the outcome measures, nor were they involved in developing plans for the design or implementation of the study. No study participants were asked to advise on interpretation or writing up of results. The results of the research will be disseminated to study participants on request, and to stakeholders and the broader public as relevant.

## Results

### Study and participant characteristics

The characteristics of the 14 included studies are shown in Table 1. Mean maternal BMI ranged from 23.0 to 28.5 kg/m^2^ across studies, and mean maternal age ranged from 25 to 31 years old. The maximum sample size from each study ranged from 356 (NFBC1966) to 190,879 (FinnGen). The number of cases ranged from 107 for miscarriage in the index pregnancy (used in multivariable regression and paternal negative control analyses) to 78,472 for breastfeeding initiation (Table 2).

### Main analyses results

**Fig. 3:**
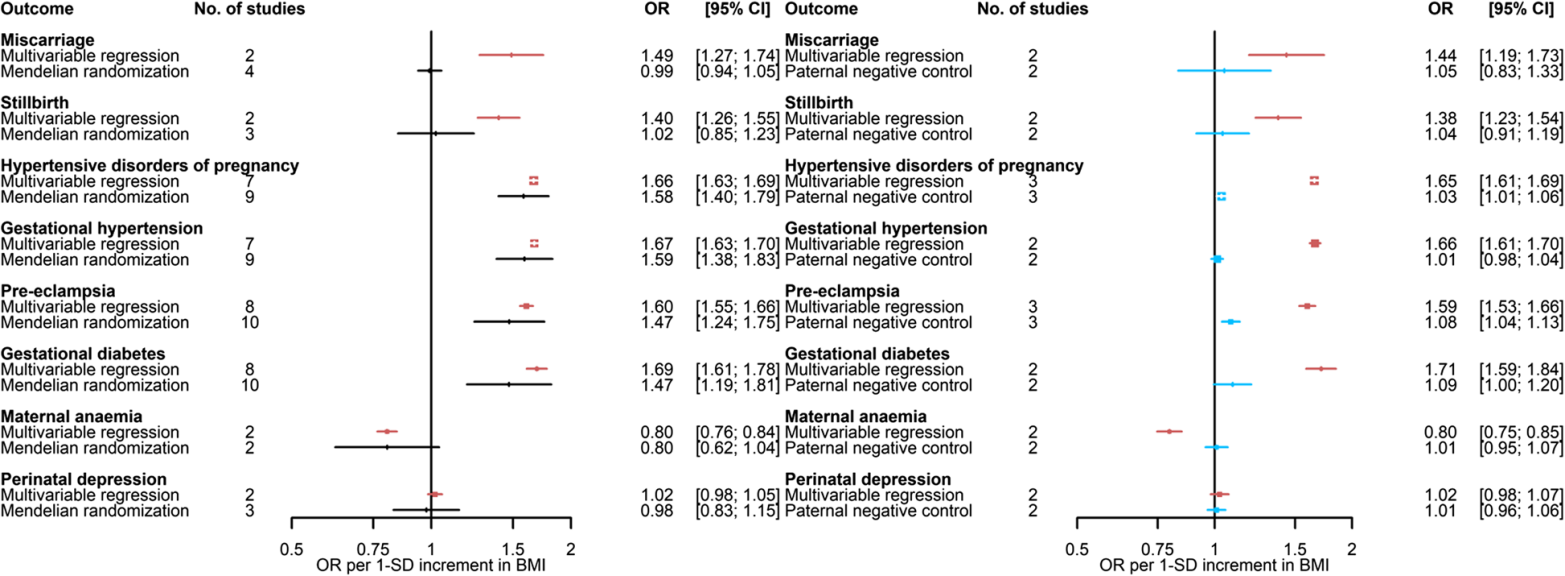
Comparison of **A** adjusted multivariable regression and main Mendelian randomisation estimates and **B** mutually adjusted multivariable regression estimates and paternal negative control (exposure, paternal body mass index)—for the association of maternal body mass index with binary outcomes (Part 1). Paternal BMI was used as a negative control exposure to explore the potential presence, direction, and magnitude of bias in multivariable estimates for associations of maternal BMI with outcomes.. Results are expressed as odds ratios per SD unit of maternal BMI and paternal BMI for ‘Multivariable regression’ and ‘Paternal negative control’, respectively. Multivariable regression results were adjusted for paternal BMI, maternal age, parity, education, smoking during pregnancy, alcohol use during pregnancy, and offspring sex where available. Paternal negative control results were adjusted for maternal BMI, paternal age, number of children (ALSPAC only), paternal education, paternal smoking, paternal alcohol use, and offspring sex. BMI, body mass index; NICU, neonatal intensive care unit

**Fig. 4:**
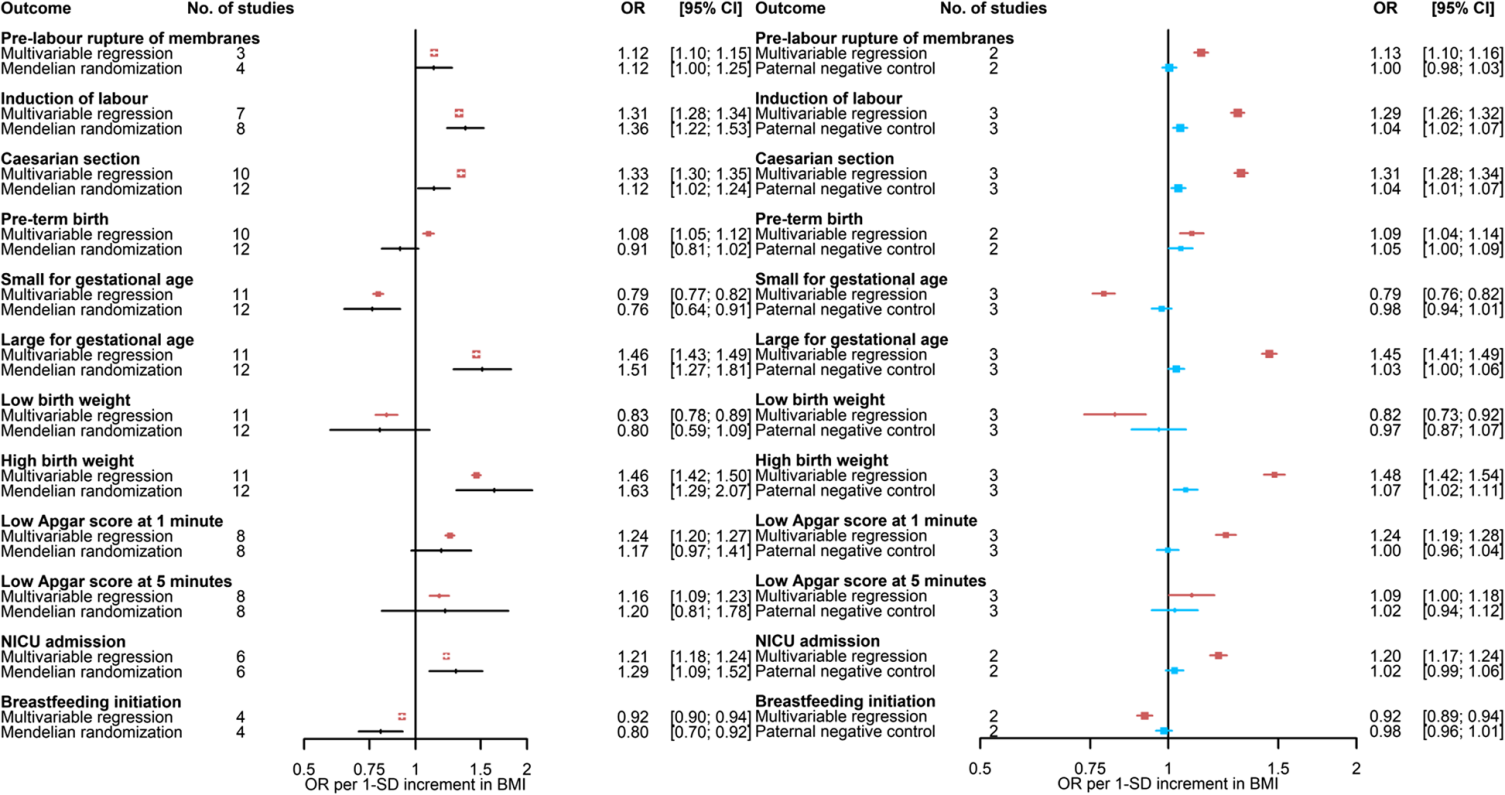
Comparison of **A** adjusted multivariable regression and main Mendelian randomisation estimates and **B** mutually adjusted multivariable regression estimates and paternal negative control (exposure, paternal body mass index)—for the association of maternal body mass index with binary outcomes (Part 2). Paternal BMI was used as a negative control exposure to explore the potential presence, direction, and magnitude of bias in multivariable estimates for associations of maternal BMI with outcomes.. Results are expressed as odds ratios per SD unit of maternal BMI and paternal BMI for ‘Multivariable regression’ and ‘Paternal negative control’, respectively. Multivariable regression results were adjusted for paternal BMI, maternal age, parity, education, smoking during pregnancy, alcohol use during pregnancy, and offspring sex where available. Paternal negative control results were adjusted for maternal BMI, paternal age, number of children (ALSPAC only), paternal education, paternal smoking, paternal alcohol use, and offspring sex. BMI, body mass index; NICU, neonatal intensive care unit

Results for the main multivariable regression (fully adjusted model) and Mendelian randomisation (IVW) analyses are shown in Figs. 3 and 4 (binary outcomes) and Additional file 3: Supplementary Fig. 1 (continuous outcomes).

In the main multivariable regression analyses, maternal BMI was associated with 19 out of the 20 binary outcomes. Higher maternal BMI was associated with a higher risk of miscarriage, stillbirth, HDP, GH, PE, GDM, pre-labour membrane rupture, induction of labour, caesarean section, PTB, LGA, high birthweight, low Apgar score at 1 min, low Apgar score at 5 min, and NICU admission. In addition, women with higher BMI were less likely to have maternal anaemia, have a baby SGA or with low birthweight, and initiate breastfeeding (Figs. 3 and 4). There was little evidence of maternal BMI being associated with the risk of perinatal depression (Fig. 3). Higher maternal BMI was associated with higher values of most continuous outcomes (i.e. birthweight, birth length, and ponderal index) (Additional file 3: Supplementary Fig. 1).

For the Mendelian randomisation analyses, we estimated that the total *R*^2^ and mean *F*-statistic for the association of SNPs with BMI were 2.7% and 36, respectively, for the set of 97 SNPs using female-specific data from the GIANT GWAS. We observed a positive correlation (*r* = 0.67) between SNP-BMI estimates from females in the GIANT GWAS and SNP-BMI (pre-/early-pregnancy) estimates pooled across participating cohorts (Additional file 3: Supplementary Fig. 2). In agreement with multivariable regression analyses, findings from Mendelian randomisation indicated that higher maternal BMI is related to higher risk of HDP, GH, PE, GDM, pre-labour membrane rupture, induction of labour, caesarean section, LGA, high birthweight, low Apgar score at 1 min, NICU admission, lower risk of having maternal anaemia, a SGA baby, lower odds of initiating breastfeeding, and not associated with perinatal depression. On the other hand, in contrast with multivariable regression analyses, Mendelian randomisation findings did not provide support for a positive association of maternal BMI with miscarriage, stillbirth, and PTB. As expected, given the lower statistical power, confidence intervals were wider for Mendelian randomisation compared to multivariable regression analyses and included the null value for some of these outcomes (Figs. 3 and 4). For two binary outcomes (i.e. low Apgar score at 5 min and low birthweight), it was less clear whether estimates from multivariable and Mendelian randomisation are in agreement given the substantial uncertainty in the latter. For most continuous outcomes (i.e. birthweight, birth length, and ponderal index), findings from Mendelian randomisation indicated that higher maternal BMI was associated with higher values of continuous outcomes in agreement with multivariable regression analyses (Additional file 3: Supplementary Fig. 1).

Paternal negative control results supported the role of maternal BMI on stillbirth, HDP, GH, PE, GDM, maternal anaemia, pre-labour membrane rupture, induction of labour, caesarean section, SGA, LGA, high birthweight, low Apgar score at 1 min, NICU admission, and breastfeeding initiation (Figs. 3 and 4). The association of paternal BMI with maternal perinatal depression was also close to the null, consistent with maternal multivariable and Mendelian randomisation results. Associations with miscarriage, PTB, low birthweight, and low Apgar score at 5 min were imprecise and/or more similar in direction and magnitude between paternal and maternal BMI analyses. Results for continuous outcomes were strongly attenuated for paternal BMI in relation to birthweight and length (Additional file 3: Supplementary Fig. 3).

### Sensitivity analyses

Overall, findings from the main multivariable regression analyses were consistent across studies (Additional file 3: Supplementary Fig. 4), when using random-effect metanalyses (Additional file 3: Supplementary Fig. 5), and with minimally adjusted models (Additional file 3: Supplementary Fig. 6). Between-study heterogeneity was substantial (i.e. Cochrane’s Q *p*-value < 0.05) for GDM, maternal anaemia, low Apgar score at 1 min, gestational age, and birthweight (Additional file 3: Supplementary table 4).

Overall, findings from the main Mendelian randomisation analyses were not driven by any individual study as indicated by the leave-one-out analyses, although in some cases removing one study resulted in attenuation and substantial imprecision of effect estimates, such as for GDM when removing FinnGen and for delivery outcomes when removing MoBa (Additional file 3: Supplementary Fig. 7). Results were similar when using fixed- or random-effect meta-analyses to pool SNP-outcome estimates across studies (Additional file 3: Supplementary Fig. 8). There was evidence of substantial SNP heterogeneity in the IVW analyses of maternal BMI with 11 out of 20 binary outcomes and 1 out of 4 continuous outcomes (Additional file 2: Supplementary table 5). Despite that, there was no clear evidence of directional pleiotropy as evidenced by the MR-Egger intercept test (except for GDM and gestational age) (Additional file 2: Supplementary table 5). Furthermore, Mendelian randomisation results were generally consistent when using different Mendelian randomisation methods (Additional file 3: Supplementary Fig. 9), although estimates from MR-Egger were imprecise for some outcomes. Effect estimates adjusting for offspring genotype were more imprecise due to the smaller sample size; however, overall, point estimates were not substantially different compared to the main analyses with a few exceptions, such as pre-labour rupture of membranes, LGA, and high birthweight, where adjusted results were attenuated (Additional file 3: Supplementary Fig. 10).

Findings from the main paternal negative control analyses were consistent between studies (Additional file 3: Supplementary Fig. 11 for maternal associations additionally adjusted for partners BMI and Additional file 3: Supplementary Fig. 12 for paternal associations) and when comparing different models (Additional file 3: Supplementary Figs. 13–15). Findings from the main multivariable regression analyses were similar when stratified by BMI taken pre-pregnancy compared to during pregnancy (Additional file 3: Supplementary Fig. 16).

## Discussion

By triangulating different analytical approaches, our findings are compatible with higher maternal BMI being causally related to 14 out of 20 pregnancy and perinatal outcomes, including a higher risk of HDP, GH, PE, GDM, pre-labour membrane rupture, induction of labour, caesarean section, LGA, high birthweight, low Apgar score at 1 min, NICU admission, and lower odds of maternal anaemia, SGA, or breastfeeding initiation. In addition, we did not find supportive evidence for a relation of maternal BMI with perinatal depression. For other outcomes, evidence is uncertain due to inconsistencies across multiple approaches (i.e. multivariable regression results for miscarriage, stillbirth, and PTB were not supported by Mendelian randomisation) or substantial imprecision in effect estimates from Mendelian randomisation (i.e. low birthweight and low Apgar score at 5 min).

Consistent with our results, a previous study using multivariable regression reported higher maternal BMI (across the whole distribution) was associated with increased risk of HDP, GDM and LGA, and reduced risk of SGA based on data from 265,270 mother–offspring pairs (samples partly overlapping with our study) [10]. In addition, there was some evidence of a non-linear association with odds of PTB, which were higher in women who were underweight or obese [10]. In agreement with these findings, a larger study (9,282,486 mother–infant pairs in the USA) focussed on offspring outcomes indicated that higher maternal BMI was associated with a higher risk of high birthweight, LGA, and low Apgar score and reported a non-linear relationship with PTB risk [76]. Other observational studies using multivariable regression have reported that maternal BMI is associated with a higher risk of stillbirths [77], induction [78], caesarean section [78], and not initiating breastfeeding [79]. Previous Mendelian randomisation studies have focused on a limited set of outcomes and are supportive of higher maternal BMI being related to higher mean offspring birthweight [4, 27, 80] (*N* ~ 9,000 to 400,000) and GDM [81] (*N* = 5485 cases and 347,856 controls).

Recent systematic reviews of randomised controlled trials (RCTs) of diet and physical activity during pregnancy (*N* range: 12,526–34,546) reported some evidence of reduced risk of GDM, LGA, and caesarean section in those randomised to the intervention, but no effect or mixed results of the intervention on HDP, PTB, and NICU admission [82–84]. Of note, these studies aimed at managing weight gain during pregnancy rather than targeting weight reduction prior to pregnancy with a modest mean difference of − 0.7 to − 1.2 kg between women in the intervention compared to those randomised to standard care. In addition, evidence for many outcomes is uncertain due to the relatively small number of cases.

Although mechanisms are not fully understood, higher maternal BMI is likely to influence a range of processes that are involved in the aetiology of some of the outcomes of interest, such as insulin resistance, endothelial dysfunction, inflammation, and susceptibility to infection [85]. In addition, maternal dysmetabolism resulting from excess adiposity has a well-recognised impact on maternal circulating nutrients, such as glucose, lipids, and amino acids, some of which can cross the placenta and influence offspring outcomes, such as growth [4, 86, 87].

### Strengths and limitations

Key strengths of this study include exploring the potential role of maternal BMI on a wide range of pregnancy and perinatal outcomes in large samples from multiple studies using different approaches. The credibility of findings from each approach relies on the plausibility of assumptions that are often not possible to verify, such as no unmeasured confounding in multivariable regression, similar confounding, selection and measurement error between paternal and maternal BMI analyses, and no confounding or horizontal pleiotropy in Mendelian randomisation. Therefore, results in agreement across approaches strengthen the evidence on the relation of maternal BMI with the outcome. Where possible, we explored the plausibility of assumptions underlying each method. In particular, we conducted extensive sensitivity analyses to explore the plausibility of the core Mendelian randomisation assumptions and found overall these did not suggest Mendelian randomisation results were driven by weak, invalid instruments or confounding by offspring genotype.

Key limitations of this study are as follows. First, despite the large scale of our study, statistical power varied across outcomes as some outcomes have lower prevalence and/or were not collected in all cohorts. Second, despite our efforts to capture the best and most homogeneous definition for outcomes across studies, this was not always possible as exemplified by GDM, for which the data collected was notably variable across studies (e.g. from self-report to medical records-derived information), and index miscarriage (which was used for multivariable regression and paternal negative control analyses but is poorly captured in birth cohorts during the early pregnancy period). Third, while we were interested in maternal pre-pregnancy BMI, only maternal weight reflecting early-/mid-pregnancy was available in four studies. Fourth, our analyses assumed a linear effects of BMI, which may not be the case for some outcomes like PTB, and were restricted to women of European ancestry given most studies had scarce data on women from other ancestries. While this reduces the risk of confounding by ethnicity or population structure, it may limit the generalisability to other populations of pregnant women.

## Conclusions

Our findings support a causal role for higher maternal BMI on a range of adverse pregnancy and perinatal outcomes. Given the high prevalence of overweight and obesity, our findings emphasise the need for development and testing of pre-conception interventions to support women maintaining a healthy BMI. This should be a key target to reduce the burden of obstetric and neonatal complications.

### Supplementary Information

**Additional file 1.** Supplementary methods. 

**Additional file 2.** Supplementary tables.

**Additional file 3.** Supplementary figures.

## Data Availability

In order to protect participant confidentiality, supporting data cannot be made openly available. Bona fide researchers can apply for access to study-specific executive committees. Summary association data for FinnGen is publicly available at https://www.finngen.fi/en/access_results. Researchers can apply for access to the UK Biobank data via the Access Management System (AMS) (https://www.ukbiobank.ac.uk/enable-your-research/apply-for-access).

## References

[1] Global Burden of Disease Collaborative Network: Global Burden of Disease Study 2019 (GBD 2019) results. In*.* Seattle, USA: Institute for Health Metrics and Evaluation (IHME); 2021.

[2] Marchi J, Berg M, Dencker A, Olander E, Begley C (2015). Risks associated with obesity in pregnancy, for the mother and baby: a systematic review of reviews. Obes Rev..

[3] Aune D, Saugstad OD, Henriksen T, Tonstad S (2014). Maternal body mass index and the risk of fetal death, stillbirth, and infant death: a systematic review and meta-analysis. JAMA..

[4] Tyrrell J, Richmond RC, Palmer TM, Feenstra B, Rangarajan J, Metrustry S, Cavadino A, Paternoster L, Armstrong LL, De Silva NMG (2016). Genetic evidence for causal relationships between maternal obesity-related traits and birth weight. JAMA.

[5] Poobalan AS, Aucott LS, Gurung T, Smith WCS, Bhattacharya S (2009). Obesity as an independent risk factor for elective and emergency caesarean delivery in nulliparous women–systematic review and meta-analysis of cohort studies. Obes Rev..

[6] Heslehurst N, Simpson H, Ells L, Rankin J, Wilkinson J, Lang R, Brown T, Summerbell C (2008). The impact of maternal BMI status on pregnancy outcomes with immediate short-term obstetric resource implications: a meta-analysis. Obes Rev..

[7] Molyneaux E, Poston L, Ashurst-Williams S, Howard LM (2014). Obesity and mental disorders during pregnancy and postpartum: a systematic review and meta-analysis. Obstet Gynecol..

[8] Molyneaux E, Pasupathy D, Kenny L, McCowan L, North R, Dekker G, Walker J, Baker PN, Poston L, Howard L (2016). Socio-economic status influences the relationship between obesity and antenatal depression: data from a prospective cohort study. J Affect Disord.

[9] Turcksin R, Bel S, Galjaard S, Devlieger R (2014). Nutrition c: Maternal obesity and breastfeeding intention, initiation, intensity and duration: a systematic review. Matern Child Nutr..

[10] Santos S, Voerman E, Amiano P, Barros H, Beilin LJ, Bergstrom A, Charles MA, Chatzi L, Chevrier C, Chrousos GP (2019). Impact of maternal body mass index and gestational weight gain on pregnancy complications: an individual participant data meta-analysis of European North American and Australian cohorts. BJOG.

[11] Lutsiv O, Mah J, Beyene J, McDonald SD (2015). The effects of morbid obesity on maternal and neonatal health outcomes: a systematic review and meta-analyses. Obes Rev.

[12] Dachew BA, Ayano G, Betts K, Alati R (2021). The impact of pre-pregnancy BMI on maternal depressive and anxiety symptoms during pregnancy and the postpartum period: A systematic review and meta-analysis. J Affect Disord.

[13] Lawlor DA, Tilling K, Davey Smith G (2016). Triangulation in aetiological epidemiology. Int J Epidemiol.

[14] Munafo MR, Davey Smith G (2018). Robust research needs many lines of evidence. Nature.

[15] Lawlor DA, Harbord RM, Sterne JA, Timpson N, Davey Smith G (2008). Mendelian randomization: using genes as instruments for making causal inferences in epidemiology. Stat Med.

[16] Smith GD, Lawlor DA, Harbord R, Timpson N, Day I, Ebrahim S (2007). Clustered environments and randomized genes: a fundamental distinction between conventional and genetic epidemiology. PLoS Med.

[17] Lipsitch M, Tchetgen Tchetgen E, Cohen T (2010). Negative controls: a tool for detecting confounding and bias in observational studies. Epidemiology.

[18] Sanderson E, Macdonald-Wallis C, Davey Smith G (2018). Negative control exposure studies in the presence of measurement error: implications for attempted effect estimate calibration. Int J Epidemiol.

[19] Ebrahim S, Davey Smith G (2008). Mendelian randomization: can genetic epidemiology help redress the failures of observational epidemiology?. Hum Genet.

[20] Smith GD, Ebrahim S (2003). ‘Mendelian randomization’: can genetic epidemiology contribute to understanding environmental determinants of disease?. Int J Epidemiol.

[21] Sanderson E, Glymour MM, Holmes MV, Kang H, Morrison J, Munafò MR, Palmer T, Schooling CM, Wallace C, Zhao Q (2022). Mendelian randomization Nature Reviews Methods Primers.

[22] Yang Q, Borges MC, Sanderson E, Magnus MC, Kilpi F, Collings PJ, Soares AL, West J, Magnus P, Wright J (2022). Associations between insomnia and pregnancy and perinatal outcomes: Evidence from mendelian randomization and multivariable regression analyses. PLoS Med.

[23] Boyd A, Golding J, Macleod J, Lawlor DA, Fraser A, Henderson J, Molloy L, Ness A, Ring S, Davey Smith G (2013). Cohort Profile: the 'children of the 90s'–the index offspring of the Avon Longitudinal Study of Parents and Children. Int J Epidemiol.

[24] Fraser A, Macdonald-Wallis C, Tilling K, Boyd A, Golding J, Davey Smith G, Henderson J, Macleod J, Molloy L, Ness A (2013). Cohort Profile: the Avon Longitudinal Study of Parents and Children: ALSPAC mothers cohort. Int J Epidemiol.

[25] Taylor K, McBride N, Goulding N, Burrows K, Mason D, Pembrey L, Yang T, Azad R, Wright J, Lawlor D: Metabolomics datasets in the Born in Bradford cohort [version 2; peer review: 1 approved, 1 approved with reservations]. Wellcome Open Res 2021, 5(264). 10.12688/wellcomeopenres.16341.2 PMC11109709 38778888

[26] Wright J, Small N, Raynor P, Tuffnell D, Bhopal R, Cameron N, Fairley L, Lawlor DA, Parslow R, Petherick ES (2013). Cohort Profile: the Born in Bradford multi-ethnic family cohort study. Int J Epidemiol.

[27] Bond TA, Richmond RC, Karhunen V, Cuellar-Partida G, Borges MC, Zuber V, Couto Alves A, Mason D, Yang TC, Gunter MJ (2022). Exploring the causal effect of maternal pregnancy adiposity on offspring adiposity: Mendelian randomisation using polygenic risk scores. BMC Med.

[28] Nohr EA, Timpson NJ, Andersen CS, Davey Smith G, Olsen J, Sorensen TI (2009). Severe obesity in young women and reproductive health: the Danish National Birth Cohort. PLoS ONE.

[29] Paternoster L, Evans DM, Nohr EA, Holst C, Gaborieau V, Brennan P, Gjesing AP, Grarup N, Witte DR, Jorgensen T (2011). Genome-wide population-based association study of extremely overweight young adults–the GOYA study. PLoS ONE.

[30] Schnurr TM, Morgen CS, Borisevich D, Beaumont RN, Engelbrechtsen L, Angquist L, Have CT, Freathy RM, Smith GD, Nohr EA (2020). The influence of transmitted and non-transmitted parental BMI-associated alleles on the risk of overweight in childhood. Sci Rep.

[31] Bliddal M, Broe A, Pottegard A, Olsen J, Langhoff-Roos J (2018). The danish medical birth register. Eur J Epidemiol.

[32] Ryckman KK, Feenstra B, Shaffer JR, Bream EN, Geller F, Feingold E, Weeks DE, Gadow E, Cosentino V, Saleme C (2012). Replication of a genome-wide association study of birth weight in preterm neonates. J Pediatr.

[33] Olsen J, Melbye M, Olsen SF, Sorensen TI, Aaby P, Andersen AM, Taxbol D, Hansen KD, Juhl M, Schow TB (2001). The Danish National Birth Cohort–its background, structure and aim. Scand J Public Health.

[34] Knight B, Shields BM, Hattersley AT (2006). The Exeter Family Study of Childhood Health (EFSOCH): study protocol and methodology. Paediatr Perinat Epidemiol.

[35] Manichaikul A, Mychaleckyj JC, Rich SS, Daly K, Sale M (2010). Chen W-MJB: Robust relationship inference in genome-wide association studies.

[36] Abraham G (2014). Inouye MJPo: Fast principal component analysis of large-scale genome-wide data.

[37] Kurki MI, Karjalainen J, Palta P, Sipilä TP, Kristiansson K, Donner K, Reeve MP, Laivuori H, Aavikko M, Kaunisto MA *et al*: FinnGen: Unique genetic insights from combining isolated population and national health register data. medRxiv 2022:2022.2003.2003.22271360.

[38] Kiiskinen T, Mars NJ, Palviainen T, Koskela J, Ramo JT, Ripatti P, Ruotsalainen S, FinnGen GC, Palotie A, Madden PAF (2020). Genomic prediction of alcohol-related morbidity and mortality. Transl Psychiatry.

[39] Magee LA, Pels A, Helewa M, Rey E, von Dadelszen P (2014). Canadian Hypertensive Disorders of Pregnancy Working G: Diagnosis, evaluation, and management of the hypertensive disorders of pregnancy: executive summary. J Obstet Gynaecol Can.

[40] Fenton TR, Kim JH (2013). A systematic review and meta-analysis to revise the Fenton growth chart for preterm infants. BMC Pediatr.

[41] Kooijman MN, Kruithof CJ, van Duijn CM, Duijts L, Franco OH (2016). van IMH, de Jongste JC, Klaver CC, van der Lugt A, Mackenbach JP *et al*: The Generation R Study: design and cohort update 2017. Eur J Epidemiol.

[42] Coolman M, de Groot CJ, Jaddoe VW, Hofman A, Raat H, Steegers EA (2010). Medical record validation of maternally reported history of preeclampsia. J Clin Epidemiol.

[43] Group HSCR (2002). The Hyperglycemia and Adverse Pregnancy Outcome (HAPO) Study. Int J Gynaecol Obstet.

[44] Metzger BE, Lowe LP, Dyer AR, Trimble ER, Chaovarindr U, Coustan DR, Hadden DR, McCance DR, Hod M, Group HSCR (2008). Hyperglycemia and adverse pregnancy outcomes. N Engl J Med.

[45] Laurie CC, Doheny KF, Mirel DB, Pugh EW, Bierut LJ, Bhangale T, Boehm F, Caporaso NE, Cornelis MC, Edenberg HJ (2010). Quality control and quality assurance in genotypic data for genome-wide association studies. Genet Epidemiol.

[46] Guxens M, Ballester F, Espada M, Fernandez MF, Grimalt JO, Ibarluzea J, Olea N, Rebagliato M, Tardon A, Torrent M (2012). Cohort Profile: the INMA–INfancia y Medio Ambiente–(Environment and Childhood) Project. Int J Epidemiol.

[47] Magnus P, Birke C, Vejrup K, Haugan A, Alsaker E, Daltveit AK, Handal M, Haugen M, Høiseth G, Knudsen GP (2016). Cohort profile update: The Norwegian mother and child cohort study (MoBa). Int J Epidemiol.

[48] Rantakallio P: Groups at risk in low birth weight infants and perinatal mortality. Acta Paediatr Scand 1969, 193:Suppl 193:191+. 4911003

[49] Nordström T, Miettunen J, Auvinen J, Ala-Mursula L, Keinänen-Kiukaanniemi S, Veijola J, Järvelin M-R, Sebert S, Männikkö M (2021). Cohort Profile: 46 years of follow-up of the Northern Finland Birth Cohort 1966 (NFBC1966). Int J Epidemiol.

[50] Sabatti C, Service SK, Hartikainen A-L, Pouta A, Ripatti S, Brodsky J, Jones CG, Zaitlen NA, Varilo T, Kaakinen M *et al*: Genome-wide association analysis of metabolic traits in a birth cohort from a founder population. *Nature Genetics* 2009, 41(1):35–46. 10.1038/ng.271 PMC2687077 19060910

[51] Järvelin MR, Hartikainen-Sorri AL, Rantakallio P (1993). Labour induction policy in hospitals of different levels of specialisation. Br J Obstet Gynaecol.

[52] Taanila A, Ebeling H, Kotimaa A, Moilanen I, Järvelin MR (2004). Is a large family a protective factor against behavioural and emotional problems at the age of 8 years?. Acta Paediatr.

[53] Mitchell R, Hemani, G., Dudding, T., Corbin, L., Harrison, S., Paternoster, L: UK Biobank Genetic Data: MRC-IEU Quality Control, version 2 - Datasets - data.bris. data.bris. 2018.

[54] Bycroft C, Freeman C, Petkova D, Band G, Elliott LT, Sharp K, Motyer A, Vukcevic D, Delaneau O, O'Connell J (2018). The UK Biobank resource with deep phenotyping and genomic data. Nature.

[55] O'Connell J, Sharp K, Shrine N, Wain L, Hall I, Tobin M, Zagury JF, Delaneau O, Marchini J (2016). Haplotype estimation for biobank-scale data sets. Nat Genet.

[56] Guillemette L, Allard C, Lacroix M, Patenaude J, Battista M-C, Doyon M, Moreau J, Ménard J, Bouchard L (2016). Ardilouze J-L: Genetics of Glucose regulation in Gestation and Growth (Gen3G): a prospective prebirth cohort of mother–child pairs in Sherbrooke Canada. BMJ Open.

[57] Magnus P, Irgens LM, Haug K, Nystad W, Skjaerven R, Stoltenberg C, MoBa Study G: Cohort profile: the Norwegian Mother and Child Cohort Study (MoBa). Int J Epidemiol 2006; 35(5):1146-1150 10.1093/ije/dyl170 16926217

[58] Sudlow C, Gallacher J, Allen N, Beral V, Burton P, Danesh J, Downey P, Elliott P, Green J (2015). Landray MJPm: UK biobank: an open access resource for identifying the causes of a wide range of complex diseases of middle and old age..

[59] StataCorp: Stata Statistical Software: Release 15. In*.*: College Station, TX: StataCorp LLC; 2017.

[60] team Rc: R: A Language and Environment ofr Statistical Computing. In*.* Edited by Computing RFfS. Vienna, Austria; 2017.

[61] Lawlor DA (2016). Commentary: Two-sample Mendelian randomization: opportunities and challenges. Int J Epidemiol.

[62] Locke AE, Kahali B, Berndt SI, Justice AE, Pers TH, Day FR, Powell C, Vedantam S, Buchkovich ML, Yang J (2015). Genetic studies of body mass index yield new insights for obesity biology. Nature.

[63] Speliotes EK, Willer CJ, Berndt SI, Monda KL, Thorleifsson G, Jackson AU, Allen HL, Lindgren CM, Luan Ja, Mägi RJNg: Association analyses of 249,796 individuals reveal 18 new loci associated with body mass index. 2010, 42(11):937. 10.1038/ng.686 PMC3014648 20935630

[64] Yengo L, Sidorenko J, Kemper KE, Zheng Z, Wood AR, Weedon MN, Frayling TM, Hirschhorn J, Yang J, Visscher PM (2018). Meta-analysis of genome-wide association studies for height and body mass index in approximately 700000 individuals of European ancestry. Hum Mol Genet.

[65] Burgess S, Davies NM, Thompson SG (2016). Bias due to participant overlap in two-sample Mendelian randomization. Genet Epidemiol.

[66] Burgess S, Thompson SG (2011). Bias in causal estimates from Mendelian randomization studies with weak instruments. Stat Med.

[67] Burgess S, Butterworth A, Thompson SG: Mendelian randomization analysis with multiple genetic variants using summarized data. Genet Epidemiol. 2013, 37(7):658-665 10.1002/gepi.21758 PMC4377079 24114802

[68] Bowden J, Davey Smith G, Burgess S (2015). Mendelian randomization with invalid instruments: effect estimation and bias detection through Egger regression. Int J Epidemiol.

[69] Bowden J, Davey Smith G, Haycock PC (2016). Burgess SJGe: Consistent estimation in Mendelian randomization with some invalid instruments using a weighted median estimator.

[70] Hartwig FP, Davey Smith G, Bowden J: Robust inference in summary data Mendelian randomization via the zero modal pleiotropy assumption. 2017, 46(6):1985-1998. 10.1093/ije/dyx102 PMC5837715 29040600

[71] Warrington NM, Beaumont RN, Horikoshi M, Day FR, Helgeland O, Laurin C, Bacelis J, Peng S, Hao K, Feenstra B (2019). Maternal and fetal genetic effects on birth weight and their relevance to cardio-metabolic risk factors. Nat Genet.

[72] Lawlor D, Richmond R, Warrington N, McMahon G, Davey Smith G, Bowden J, Evans DM (2017). Using Mendelian randomization to determine causal effects of maternal pregnancy (intrauterine) exposures on offspring outcomes: Sources of bias and methods for assessing them. Wellcome Open Res.

[73] Evans DM, Moen GH, Hwang LD, Lawlor DA, Warrington NM (2019). Elucidating the role of maternal environmental exposures on offspring health and disease using two-sample Mendelian randomization. Int J Epidemiol.

[74] Brand JS, Gaillard R, West J, McEachan RRC, Wright J, Voerman E, Felix JF, Tilling K, Lawlor DA (2019). Associations of maternal quitting, reducing, and continuing smoking during pregnancy with longitudinal fetal growth: Findings from Mendelian randomization and parental negative control studies. PLoS Med.

[75] Madley-Dowd P, Rai D, Zammit S, Heron J (2020). Simulations and directed acyclic graphs explained why assortative mating biases the prenatal negative control design. J Clin Epidemiol.

[76] Zong X, Wang H, Yang L, Guo Y, Zhao M, Magnussen CG, Xi B (2022). Maternal pre-pregnancy body mass index categories and infant birth outcomes: A population-based study of 9 Million mother-infant pairs. Front Nutr.

[77] Aune D, Saugstad OD, Henriksen T, Tonstad S (2014). Maternal body mass index and the risk of fetal death, stillbirth, and infant death: a systematic review and meta-analysis. JAMA.

[78] Ellis JA, Brown CM, Barger B, Carlson NS (2019). Influence of maternal obesity on labor induction: a systematic review and meta-analysis. J Midwifery Womens Health.

[79] Ramji N, Quinlan J, Murphy P, Crane JM (2016). The impact of maternal obesity on breastfeeding. J Obstet Gynaecol Can.

[80] Thompson W, Beaumont R, Kuang A, Warrington N, Ji Y, Tyrrell J, Wood A, Scholtens D, Knight B, Evans D *et al*: Higher maternal adiposity reduces offspring birth weight if associated with a metabolically favourable profile. *medRxiv* 2020:2020.2005.2025.20112441. 10.1007/s00125-021-05570-9 PMC8563674 34542646

[81] Pervjakova N, Moen GH, Borges MC, Ferreira T, Cook JP, Allard C, Beaumont RN, Canouil M, Hatem G, Heiskala A *et al*: Multi-ancestry genome-wide association study of gestational diabetes mellitus highlights genetic links with type 2 diabetes. *Hum Mol Genet* 2022. 10.1093/hmg/ddac050 PMC9523562 35220425

[82] International Weight Management in Pregnancy Collaborative G: Effect of diet and physical activity based interventions in pregnancy on gestational weight gain and pregnancy outcomes: meta-analysis of individual participant data from randomised trials. *BMJ* 2017, 358:j3119. 10.1136/bmj.j3119 PMC6887834 28724518

[83] Cantor AG, Jungbauer RM, McDonagh M, Blazina I, Marshall NE, Weeks C, Fu R, LeBlanc ES, Chou R (2021). Counseling and behavioral interventions for healthy weight and weight gain in pregnancy: evidence report and systematic review for the US Preventive Services Task Force. JAMA.

[84] Teede HJ, Bailey C, Moran LJ, Bahri Khomami M, Enticott J, Ranasinha S, Rogozinska E, Skouteris H, Boyle JA, Thangaratinam S (2022). Association of antenatal diet and physical activity-based interventions with gestational weight gain and pregnancy outcomes: a systematic review and meta-analysis. JAMA Intern Med.

[85] Catalano PM, Shankar K (2017). Obesity and pregnancy: mechanisms of short term and long term adverse consequences for mother and child. BMJ.

[86] Barry C-JS, Lawlor DA, Shapland CY, Sanderson E, Borges MC: Using Mendelian randomisation to prioritise candidate maternal metabolic traits influencing offspring birthweight. *Metabolites* 2022, 12(6):537. 10.3390/metabo12060537 PMC9231269 35736469

[87] Zhao J, Stewart ID, Baird D, Mason D, Wright J, Zheng J, Gaunt TR, Evans DM, Freathy RM, Langenberg C *et al*: Causal effects of maternal circulating amino acids on offspring birthweight: a Mendelian randomisation study. *medRxiv* 2022:2022.2004.2015.22273911. 10.1016/j.ebiom.2023.104441 PMC9879767 36696816

